# Exploring Key Factors Affecting the Encapsulation Efficiency of Ligusticum Chuanxiong–Vinegar Cyperus Rotundus Essential Oil Based on QbD Principles

**DOI:** 10.3390/pharmaceutics18030393

**Published:** 2026-03-22

**Authors:** Zhongcheng Tang, Wenting Chen, Ting Zhang, Yu He, Haitong Wan

**Affiliations:** 1 School of Basic Medicine Sciences, Zhejiang Chinese Medical University, Hangzhou 310053, China; 13268256652@163.com (Z.T.); 18537943350@163.com (W.C.); 2 Zhejiang Key Laboratory of Chinese Medicine for Cardiovascular and Cerebrovascular Disease, Hangzhou 310053, China; 3 School of Pharmaceutical Sciences, Zhejiang Chinese Medical University, Hangzhou 310053, China; zhangting55@zcmu.edu.cn (T.Z.); heyu0923@hotmail.com (Y.H.); 4 Academy of Chinese Medical Sciences, Henan University of Chinese Medicine, Zhengzhou 450046, China

**Keywords:** Quality by Design, Chuanxiong–Vinegar cyperus rotundus volatile oil, inclusion process, factorial design

## Abstract

**Objective**: The objective is to investigate and optimize the β-cyclodextrin inclusion process for volatile oils in Ligusticum Chuanxiong–Vinegar cyperus rotundus based on Quality by Design (QbD) principles. **Methods**: First, ligustilide and α-cyperone were selected as inclusion process indicator components using high-performance liquid chromatography–tandem mass spectrometry (HPLC-MS). Single-factor experiments were conducted to preselect the inclusion speed based on ligustilide and α-cyperone content as evaluation criteria. Subsequently, using the inclusion rates of ligustilide and α-cyperone as evaluation criteria, a factorial design was employed to investigate the inclusion temperature, inclusion time, and the volume ratio of β-cyclodextrin solution to essential oil, thereby optimizing the inclusion process parameters. Finally, the inclusion process parameters were validated, and the inclusion rates were determined. The obtained inclusion complexes were characterized by microscopic analysis, Fourier-transform infrared spectroscopy (FT-IR), X-ray diffraction analysis (XRD), and differential scanning calorimetry (DSC). Furthermore, phase dissolution studies and molecular docking were employed for confirmation. **Results**: The optimal process parameters were determined as follows: encapsulation speed of 300 rpm, β-cyclodextrin solution excess of 6, encapsulation time of 2.5~3 h, and encapsulation temperature of 30~35 °C. The encapsulation rates for ligustilide and α-cyperone in the resulting inclusion complex were 63.15~64.74% and 71.33~76.89%, respectively. Structural characterization confirmed the formation of the inclusion complex. **Conclusions**: This inclusion process is reliable and provides a reference for preparing β-cyclodextrin inclusion complexes of volatile oils in formulations containing the Chuanxiong–Vinegar cyperus rotundus drug pair.

## 1. Introduction

Both Chuanxiong and Vinegar cyperus rotundus volatile oils are primarily composed of ligustilide, butylphthalide, α-cyperone, and cyperenone, among others [1,2,3,4]. These compounds exhibit analgesic, anti-inflammatory, antioxidant, and antidepressant effects [5,6,7], contributing to their pain-relieving efficacy. However, most volatile oil components suffer from susceptibility to oxidation and volatility [8], making them unsuitable for long-term storage. Cyclodextrin inclusion technology is a commonly used method to improve certain undesirable properties in drugs and enhance their stability. Experiments have demonstrated that β-cyclodextrin can be used for the inclusion of volatile oils in medicinal materials such as Chuanxiong and Vinegar cyperus rotundus [9,10]. Therefore, based on the extraction process of Chuanxiong–Vinegar cyperus rotundus, preparing β-cyclodextrin inclusion complexes of its volatile oils enables more complete retention of volatile components in formulations, thereby better exerting therapeutic effects.

The concept of Quality by Design (QbD) was first proposed in 1985 by renowned modern quality management expert Juran [11] and adopted by the U.S. Food and Drug Administration (FDA) for pharmaceutical regulation in 2004. Subsequently, the International Conference on Harmonization of Technical Requirements for Registration of Pharmaceuticals for Human Use (ICH) defined QbD in its Q8 guideline document. This approach begins with the target product’s quality as the starting point for R&D, designs scientifically sound experiments, deeply understands product attributes and controls processes, investigates the relationships between product/raw material quality attributes and process parameters, establishes a robust design space for the process using mathematical models, and implements validation and quality risk management [12,13]. Guided by QbD principles, Design of Experiments (DOE) is frequently employed to design and control experimental factors and response variables. This approach maximizes information acquisition with minimal experimental runs, enabling scientific conclusions and enhancing experimental efficiency and accuracy [14,15]. The factorial design selected for this experiment is a type of DOE experimental design. It not only accurately estimates the magnitude of the main effects of each experimental factor but also estimates the magnitude of interaction effects between factors at various levels. Compared to other experimental methods, it effectively avoids complex experimental combinations and one-sided experimental results, yielding more comprehensive, flexible, and systematic experimental combinations [16,17]. Currently, the integration of QbD principles with DOE is widely applied across various stages of new drug development, including process route studies [18,19,20], analytical method development [21,22,23,24], and the establishment of quality standards [25,26,27]. This approach enhances the efficiency of drug development while ensuring pharmaceutical quality control [12].

This study first identified the volatile oil components of Chuanxiong–Vinegar cyperus rotundus using high-performance liquid chromatography–mass spectrometry (HPLC-MS). Subsequently, guided by QbD principles, factorial design experiments were employed to explore, optimize, and validate the preparation of β-cyclodextrin inclusion complexes from this volatile oil. Key factors examined included inclusion time, inclusion temperature, and β-cyclodextrin molar ratio [9,28,29] to generate a design space, providing scientifically informed selection for optimal inclusion complex preparation conditions. Finally, physical characteristics of the inclusion complexes were confirmed via Fourier-transform infrared spectroscopy (FT-IR) analysis and other methods.

## 2. Materials and Methods

### 2.1. Materials

#### 2.1.1. Instruments

The instruments used include the following: Agilent 1260 infinity iii high-performance liquid chromatograph equipped with G7180A InfinityLab Assist Hub, G7111A 1260 Quat Pump VL, G7129A 1260 Vialsampler, G7116A 1260 MCT, G7115A 1260 DAD WR, and Chemstation workstation system (Agilent Technologies, Inc., Santa Clara, CA, USA); SYNAPT G2-Si Ultra-Performance Liquid Chromatography–Quadrupole Time-of-Flight Mass Spectrometry (UPLC-Q-TOF/MS) (Waters Corporation, Milford, MA, USA); Thermo Fisher Nicolet iS50 Far-Infrared to Visible Spectrometer (Thermo Fisher Scientific Inc., Waltham, MA, USA); XRD-6100 X-ray Diffractometer (Shimadzu Corporation, Kyoto, Japan); KQ5200DE CNC Ultrasonic Cleaner (Kunshan Ultrasonic Instruments Co., Ltd., Kunshan, Jiangsu, China); XP105 Microbalance (1/100,000 precision) (Mettler Toledo, Greifensee, Switzerland); IS1000-2C Electronic Balance (0.01 g–1100 g) (Esmate Technology Co., Ltd., Shenzhen, Guangdong, China); Model 84-1 Magnetic Stirrer (Shanghai Meiyingpu Instrument Manufacturing Co., Ltd., Shanghai, China); and DSC 3500 Sirius(NETZSCH, Selb, Bavaria, Germany).

#### 2.1.2. Reagent

The reagent used includes the following: α-Cyperone (China National Institute for Food and Drug Control, Beijing, China, Batch No.: 110748-202318, Specification: 0.15 mL, Purity: 98.5%); Ligustilide (China National Institute for Food and Drug Control, Beijing, China, Batch No.: 111737-202311, Specification: 20 μL/vial, Purity: 98.0%); β-Cyclodextrin (Anhui Shanhe Pharmaceutical Excipients Co., Ltd., Batch No.: 201223, Specification: 2 kg/bag); Acetonitrile (Shanghai Aladdin Bio-Chem Technology Co., Ltd., Shanghai, China, Chromatography Grade, Purity ≥ 99.9%); Phosphoric Acid (Shanghai Aladdin Bio-Chem Technology Co., Ltd., Chromatography Grade, Purity: 85–90%); Methanol (Shanghai Aladdin Bio-Chem Technology Co., Ltd., Chromatography Grade, Purity ≥ 99.9%); and Purified Water (Wahaha Purified Water Co., Ltd., Hangzhou, Zhejiang, China).

Vinegar cyperus rotundus (East China Pharmaceutical Co., Ltd., Hangzhou, Zhejiang, China, Batch: 20240531), Ligusticum Chuanxiong Hort (East China Pharmaceutical Co., Ltd., Hangzhou, Zhejiang, China, Batches: 20240729, 20240819), identified according to the 2020 edition of the Chinese Pharmacopeia by Traditional Chinese Medicine Preparation Laboratory, Institute of Traditional Chinese Medicine for Cardiovascular and Cerebrovascular Diseases, Zhejiang University of Chinese Medicine. Sample certificates were stored at the Traditional Chinese Medicine Preparation Laboratory, Institute of Traditional Chinese Medicine for Cardiovascular and Cerebrovascular Diseases, Zhejiang University of Chinese Medicine.

### 2.2. Methods

#### 2.2.1. Preparation of Inclusion Complexes

A total of 60 g of Chuanxiong (Ligusticum Chuanxiong) and 18 g of Vinegar cyperus rotundus were weighed. Five times the amount of water was added, and the mixture was soaked for 1 h. The temperature was then adjusted to maintain a gentle boil for 9.5 h. The volatile oil and aqueous solution were collected from the collector. The collected solution was diluted with anhydrous ethanol to a final volume of 100 mL, resulting in a clear, transparent volatile oil solution.

A certain amount of β-cyclodextrin was weighed, and 10 times its weight of water was added. The mixture was stirred at 60 °C until complete dissolution was achieved, resulting in a saturated β-cyclodextrin solution. A total of 5 mL of volatile oil ethanol solution was taken and injected into the saturated β-cyclodextrin solution. After stirring at a constant temperature for the specified time, the mixture was sealed and refrigerated for 24 h. The mixture was filtered, and the filter cake was washed with an appropriate amount of pure water and anhydrous ethanol until no residual volatile oil odor remained. Then, it was air-dried at 25 °C to obtain the inclusion compound powder [9,30].

#### 2.2.2. Liquid Chromatography–Mass Spectrometry Conditions

The liquid chromatography–mass spectrometry (LC-MS) technique was selected to determine and analyze the components in the volatile oil. The LC-MS conditions are as follows.

Chromatography conditions: A Waters CORTECS UPLC T3 column (2.1 mm × 100 mm, 1.6 µm, Waters Corporation, Milford, MA, USA) was used at a column temperature of 30 °C and a flow rate of 0.3 mL/min. The mobile phase is acetonitrile (A) with 0.1% formic acid water (B), with a gradient elution over a period of 0–38 min (0–2 min, 5% A; 2–3 min, 5–27% A; 3–11 min, 27–49% A; 11–35 min, 49–94% A; 35–37 min, 94–100% A; 37–38 min, 100% A); the sample temperature was 10 °C and the injection volume was 2 μL.

Mass spectrometry conditions: The ion source employed electrospray ionization (ESI), and scans were conducted in both positive ion mode and negative ion mode. The mass spectrometry scanning mode was continuous full scan, with a scan time of 0.2 s, within the *m*/*z* range of 50–1200. The capillary voltage was 3.0 kV for positive ions and 2.5 kV for negative ions. The sample cone voltage was 40 V. The source bias was 80 V. The ion source temperature was 120 °C. The desolvation temperatures were 500 °C for positive ions and 400 °C for negative ions. The desolvation flow rates were 1000 L/h for positive ions and 800 L/h for negative ions. The nebulizer gas pressure was 6.5 bar. The low collision energy was 6 volts, and the high collision energy ranged from 15 to 45 volts. Mass spectrometer calibration was performed using sodium formate, with real-time mass calibration via leucine enkephalin (positive ion mode: *m*/*z* 556.2771, negative ion mode: *m*/*z* 554.2615).

#### 2.2.3. Inclusion Complex Evaluation Method

High-performance liquid chromatography (HPLC) was employed to determine inclusion efficiency and evaluate the inclusion process. The HPLC conditions are as follows.

Chromatographic conditions: A Welch Ultimate Plus C_18_ column (4.6 mm × 250 mm, 5 μm) was used at a column temperature of 30 °C and a flow rate of 1.0 mL/min. Detection wavelengths were 254 nm and 280 nm, with a reference wavelength of 360 nm. The mobile phase was acetonitrile: 0.1% phosphoric acid aqueous solution = 60:40, eluted isodromically.

Preparation of test solution: A certain amount of the complex compound was taken and precisely weighed. It was placed in a stoppered conical flask, and an appropriate amount of anhydrous ethanol was precisely added to reach the required volume. The flask was weighed, and then ultrasonicated (power 25 W, frequency 40 kHz) for 0.5 h. After the solution cooled down, it was reweighed, and the weight loss was replenished with anhydrous ethanol. Then, the solution was centrifuged (5000 r/min) for 5 min and filtered, and the filtrate was collected, thus obtaining the test solution.

Preparation of standard solutions: The ligustilide standard and α-cyperone standard were separately precisely weighed, dissolved in methanol I, and then prepared into standard solutions with concentrations of 195.8 μg/mL and 178.0 μg/mL, respectively. These two standard solutions were precisely aspirated in a 3:2 ratio to obtain a mixed standard solution, in which the concentration of ligustilide was 117.5 μg/mL, and the α-cyperone concentration was 71.2 μg/mL.

Sequentially, 10 μL of the mixed standard solution, the volatile oil solution, and the test sample solution were precisely aspirated, respectively. The content was determined according to the “chromatographic conditions”, and the inclusion rate was calculated using the following formula:

Rate (%) = Mass of Component in Encapsulate/Mass of Component in Volatile Oil Input × 100%

#### 2.2.4. The Determination of Critical Quality Attribute (CQAs)

This study aims to identify and control the key factors influencing the encapsulation process, thereby obtaining the optimal encapsulation method. Among the evaluation indicators of inclusion compounds, the most direct and important ones are inclusion rate and drug loading capacity. During the process of extracting the volatile oil of Ligusticum Chuanxiong by steam distillation, emulsification often occurs due to the slow speed of oil–water separation, making it impossible to accurately read the volume of the volatile oil. Therefore, in this study, the entrapment rate of the volatile oil of Ligusticum Chuanxiong—Vinegar cyperus rotundus could not be selected as a CQA. Referring to the volatile oil components that need to be identified under the identification items of Ligusticum Chuanxiong and Vinegar cyperus rotundus in the 2025 edition of the Chinese Pharmacopeia, ligustilide was respectively selected as the representative component of Ligusticum Chuanxiong volatile oil, and α-cyperidone was selected as the representative component of Vinegar cyperus rotundus volatile oil. In conclusion, the CQAs in this study were the inclusion rates of ligustilide and α-cyperidone.

#### 2.2.5. Screening of Critical Process Parameters (CPPs)

**Figure 1:**
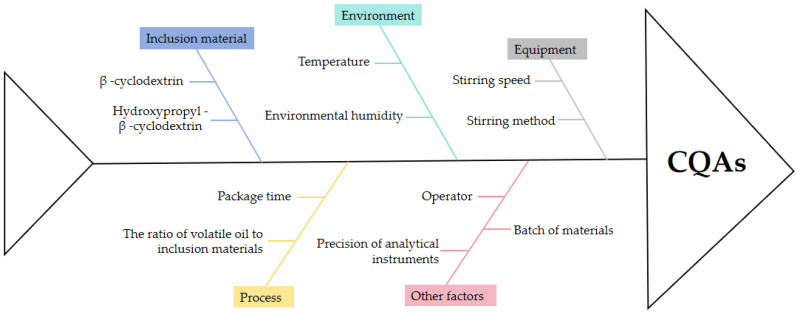
The fishbone diagram is used to identify potential key process parameters.

CPPs are some process parameters that have significant and important impacts on product quality, and their determination requires risk assessment. In this study, the Ishikawa diagram was used as a risk identification tool to screen the CPPs in the process [31]. As shown in Figure 1, the potential critical method parameters (PCMPs) mainly come from encapsulation time, encapsulation temperature, the ratio of volatile oil to β-cyclodextrin, stirring speed, and other factors. The RPN for each PCMP was calculated based on severity (S), occurrence rate (O), and difficulty measurement (D) (RPN = S × O × D; low risk level: RPN < 50; medium-risk level: 50 ≤ RPN < 125; high-risk level: RPN ≥ 125). The higher the RPN score, the greater the risk of the PCMP. The value range for each indicator was set from 1 to 10, and the results are shown in Table 1.

According to Table 1, the high-risk level factors (volatile oils to β-cyclodextrin, the temperature, and the inclusion time) were selected as the CPPs. Through factorial design experiments, the influence of CPPs on CQAs was analyzed, and the operational design region (MODR) was obtained. At the same time, the medium-risk factor (stirring speed) was screened and controlled.

#### 2.2.6. Study on the Preparation Process of Incorporated Compounds

##### Investigation of Stirring Speed

A specific volume of saturated β-cyclodextrin solution was prepared. Under the condition that the volume ratio of the β-cyclodextrin solution to the ethanol solution of the volatile oil was 6, at room temperature, with the inclusion time set to 1 h, the inclusion efficiency was measured at inclusion speeds of 300 rpm and 800 rpm (Table 2).

##### Factorial Design Experiment to Investigate CPPs

Based on the encapsulation conditions of volatile oils in traditional Chinese medicine granules and the preliminary experimental results, the influence degree of CPPs on CQAs was evaluated by using 2^3^ factor design. Firstly, the level tables of 3 factors were determined, as shown in Table 3. Then, 8 experiments were generated using Design-Expert^®^ (software version 12), and they were repeated within groups (n = 2). The experimental combinations are shown in Table 4. Linear regression and equation fitting were conducted on the inclusion rate of Ligustilide and α-Cyperone to determine the fitting degree of the designed model. Finally, the influence of each factor on the inclusion rate of Ligustilide and α-Cyperone was evaluated, and the operation design region (MODR) of the method was ultimately analyzed and generated.

#### 2.2.7. Characterization of the Inclusion Compound

##### Appearance

A certain amount of saturated β-cyclodextrin solution was taken, and the essential oil was added to it (without stirring or heating). Then, it was sealed and refrigerated for 24 h. The mixture was filtered, and the filter cake was rinsed with an appropriate amount of anhydrous ethanol 1–2 times. The β-cyclodextrin and essential oil physical mixture was obtained by air-drying at room temperature. The β-cyclodextrin, the physical mixture, and the inclusion compound were weighed, respectively, and placed in a dish. The appearance of these three substances was observed and compared.

##### Microscopic Analysis

First, the volatile oil was separated from the inclusion compound and β-cyclodextrin powder through sieve No. 4. Then, a small amount of the sample was placed on a glass slide, pure water was added to make a glass slide sample, a cover glass was placed on top, and finally, it was observed under an optical microscope.

##### Fourier-Transform Infrared Spectroscopy (FT-IR) Analysis

First, an appropriate amount of KBr powder was added to the dry powders of the inclusion complexes, physical mixtures, and β-cyclodextrin samples, and they were thoroughly mixed. Then, they were pressed into pellets. Finally, infrared spectra were scanned in the range of 400–4000 cm^−1^ with a resolution of 4 cm^−1^. The scanning signals were repeated 32 times, and the spectra were recorded.

##### X-Ray Diffraction (XRD) Analysis

The dry powders of inclusion complexes, physical mixtures, and β-cyclodextrin samples were placed in the X-ray holder. The radiation wavelength was set to Cu-κα (λ = 1.5406 × 10^−10^ m), the voltage was 40 kV, the current was 30 mA, the scanning range was 5–80°, the scanning rate was 4°/min, and the preset time was set to 30 s.

##### Differential Scanning Calorimetry (DSC)

The complexation of drug molecules with β-cyclodextrin molecules is a process driven by both enthalpy and entropy. The disappearance of the endothermic peak, the broadening of the peak shape, the shift in the peak, or the generation of a new peak indicate changes in the lattice, melting point, boiling point, sublimation point, etc. The inclusion compounds, physical mixtures, and β-cyclodextrin samples were prepared and placed in an aluminum crucible. Nitrogen gas was used as the protective gas with a flow rate of 40 mL/min. The heating rate was 10 °C/min, and the heating range was 35–200 °C.

##### Phase-Solubility Studies

β-cyclodextrin aqueous solutions with concentrations of 0, 2, 4, 6, 8, and 10 mmol/L were prepared. Three portions of each concentration solution were taken and placed in test tubes, each containing 10 mL. Excess volatile oil was added to each solution at 25, 35, and 45 °C, and the tubes were ultrasonically shaken for 5 min. Then, they were left at each temperature for 3 days. The contents were filtered and measured, and the concentrations of ligustilide and α-cyperidone were calculated. The dissolution curves of the complexes were plotted with the concentration of β-cyclodextrin as the abscissa and the concentrations of each substance at 25, 35, and 45 °C as the ordinate. The inclusion constant (*Kc*) of the complexes was calculated using the following formula, where the intercept represents the solubility of each substance in the aqueous solution without adding β-cyclodextrin:

Kc = k/b(1−k)

Here, *k* represents the slope; b represents the intercept.

Thermodynamic parameters were related through the van’t Hoff equation [32,33]:

lnK=−ΔH/R∗1/T+ΔS/R

ΔG=−RTlnK=ΔH−TΔS

##### Molecular Docking

The ligustilide and α-cyperidone were selected as ligand molecules, and β-cyclodextrin was chosen as the receptor molecule for docking. The interaction between these two components and β-cyclodextrin was investigated, and the most likely conformation for the formation of cyclodextrin inclusion complexes was determined through calculation. In this study, the 3D molecular structure of β-cyclodextrin was downloaded from the Cambridge Crystallographic Database (http://www.ccdc.cam.ac.uk/, accessed on 4 February 2026), and the 3D molecular structures of ligustilide and α-cyperone were downloaded from the PubChem database (https://pubchem.ncbi.nlm.nih.gov/, accessed on 4 February 2026) in sdf format. The above two ligand molecules were respectively subjected to hydrogenation, torsion bond detection, and centering pre-processing. The box size for docking was set to 60 × 60, the simulation times were 50, and the AutoDock running parameters and calculation methods were prepared. Finally, the binding energy and the number of hydrogen bonds generated at the docking position could be obtained through AutoDock (software version 4.2) [33].

## 3. Result

### 3.1. Essential Oil Component Analysis

**Figure 2:**
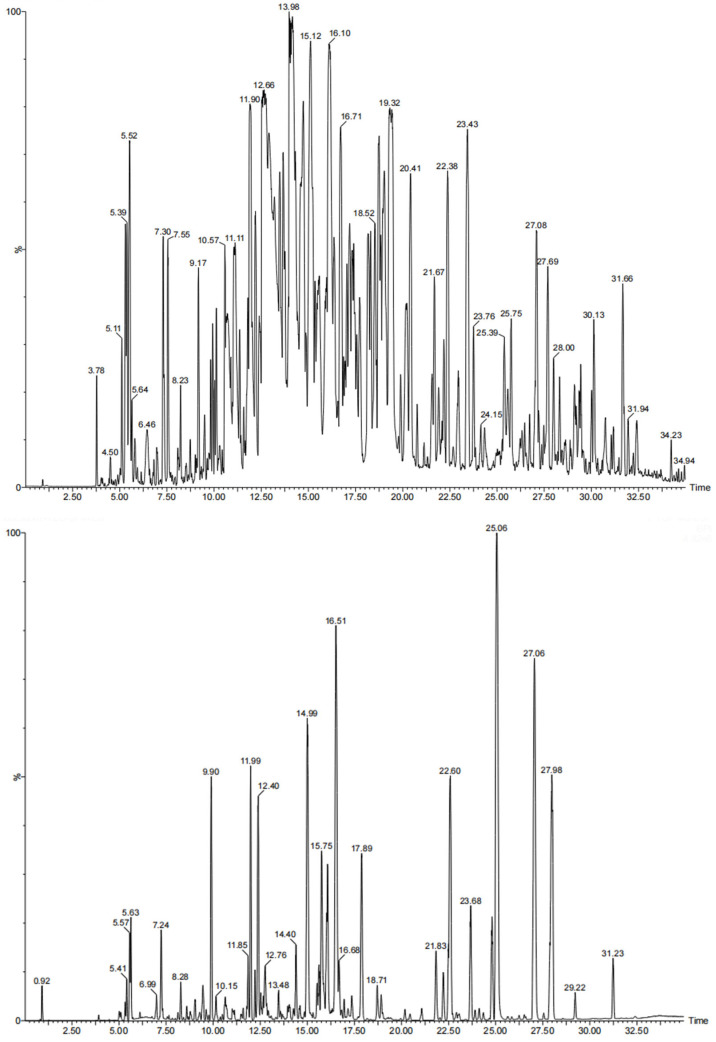
Primary base peak ionization spectra of positive and negative ion modes for Chuanxiong–Vinegar cyperus essential oil.

The base peak ionization (BPI) spectra in the positive ion mode and negative ion mode are shown in Figure 2. Through systematic analysis of the parent ion data in the primary HPLC-MS spectra and secondary spectral data, a total of 55 major chemical components were finally identified, including 12 phenylacetone monomer compounds, seven phenylacetone dimer compounds, five monoterpenoid compounds, nine sesquiterpenoid compounds, seven fatty acid compounds, two fatty acid ester compounds, nine aromatic compounds, and four other compounds. The details are summarized in Table 5.

Based on HPLC-MS analysis and a literature review [34], and for convenience in subsequent experimental measurements, ligustilide and α-cyperone—both with relatively high content and known pharmacological effects—were selected as representative components of Chuanxiong and Vinegar cyperus volatile oils, respectively, to evaluate oil inclusion rates. In subsequent factorial design experiments, liquid chromatography was employed for quantifying these two components.

### 3.2. Investigation of Combined Rotational Speed

A single-factor preliminary experiment was conducted to evaluate inclusion rotation speed. While maintaining consistent conditions, experiments were performed at the highest and lowest rotation speeds. Results are shown in Table 6.

Based on the content of ligustilide and α-cyperone, the difference in inclusion rotation speed did not significantly affect the component content in the inclusion complex (*p* > 0.05). Therefore, within the range of 300–800 rpm, 300 rpm was selected as the inclusion rotation speed for the factorial design experiment.

### 3.3. Optimization of Inclusion Process via Factorial Design Experiment

The data obtained from the factorial design experiment (Table 7) were subjected to multiple regression fitting, and the analysis of variance was presented in Table 8 and Table 9. We know from this that the simulation fitting degrees of ligustilide inclusion rate and α-cyperidone inclusion rate were both good. The ratio of β-cyclodextrin solution to volatile oil volume and inclusion temperature had a significant effect on the ligustilide inclusion rate (*p* < 0.01), and the interaction between inclusion time and inclusion temperature also had a significant effect on the ligustilide inclusion rate (*p* < 0.01). The ratio of the volume of β-cyclodextrin solution to that of volatile oil had a significant effect on the inclusion rate of α-cyperazone (*p* < 0.01), and the interaction between the inclusion time and the inclusion temperature had a relatively significant effect on the inclusion rate of α-cyperazone (*p* < 0.05).

**Figure 3:**
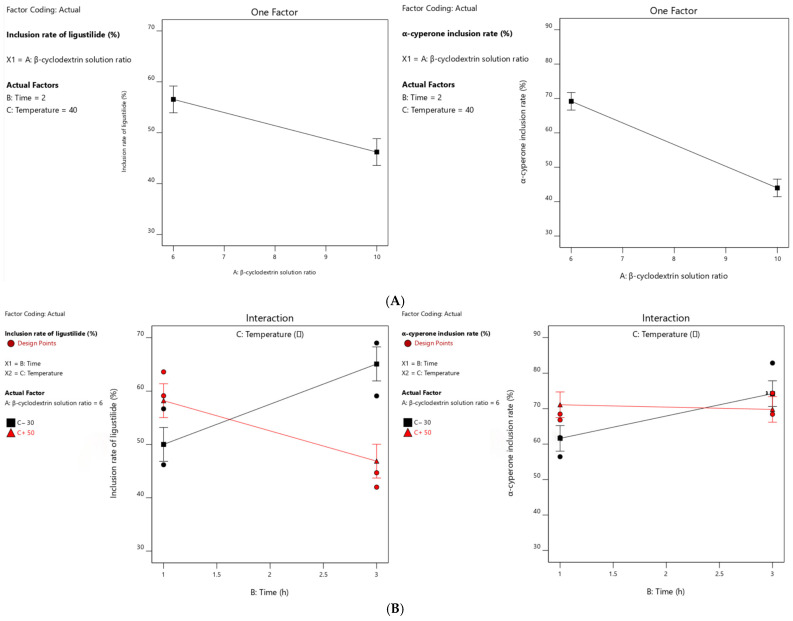
Influence trend of (**A**) β-cyclodextrin volume ratio on inclusion rate and (**B**) interaction effect of inclusion time and temperature on Ligustilide inclusion rate.

The doubling of β-cyclodextrin was a significant influencing factor in this experiment (*p* < 0.01), and thus, the analysis results of this factor are shown in Figure 3A. These results suggested that the doubling of β-cyclodextrin is negatively correlated with the inclusion rate of this experiment. Therefore, a doubling of β-cyclodextrin to 6 was selected as one of the optimal conditions for this experiment.

After choosing the doubling of β-cyclodextrin as 6, the influence of the interaction between inclusion temperature and inclusion time on the inclusion rate was shown in Figure 3B. These results suggest that the inclusion rate of ligustilide decreases with the increase in temperature, while the inclusion rate and temperature of α-cyperidone increase with the increase in inclusion time.

The data were subjected to a normality test using GraphPad (8.0.2). After the Shapiro–Wilk normality test, the results showed that they conformed to a normal distribution (Table 10). Therefore, a variance analysis was conducted for each group of data, with the minimum expectation being the mean + standard deviation; that is, the encapsulation rate of ligustilide was greater than 61.12%, and the encapsulation rate of α-cyperidone was greater than 71.12%. The following design space was obtained [35].

### 3.4. Validation of Design Space and Inclusion Rate Determination

**Figure 4:**
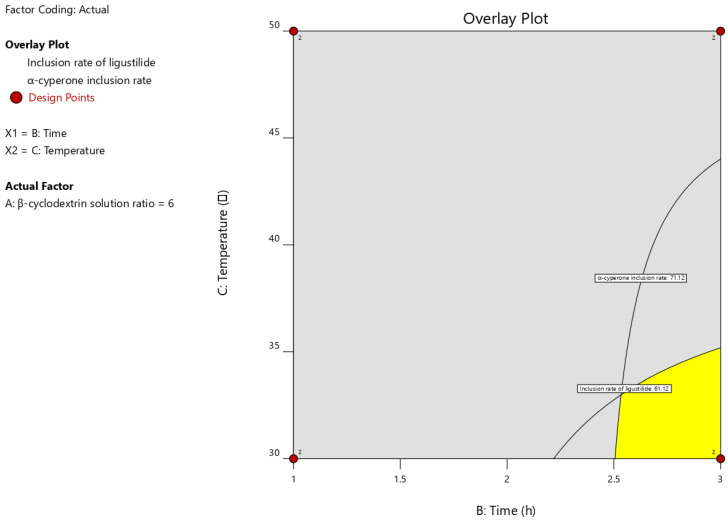
Design space obtained when the inclusion ratio of ligustilide is ≥61.12% and that of α-cyperone is ≥71.12% at a β-cyclodextrin volume ratio of 6. The grey area indicates the high-risk zone of the process, the yellow area represents the stable zone, and the red dots represent the design points. The number “2” near the red dots indicates that two experiments were conducted at this design point.

To verify the correctness of the spatial prediction in the design shown in Figure 4, we randomly selected multiple points within this interval for verification. The conditions it includes are shown in the Table below. Additionally, two sets of parallel experiments were conducted under each condition, and the 95% confidence intervals for each condition of the model are also shown in Table 11.

As shown in Table 11, the average inclusion rates of Ligustilide and α-Cyperone in each group of verification experiments were all within the confidence interval, which indicates that the model is reliable and the condition space is also credible.

#### 3.4.1. Appearance

**Figure 5:**
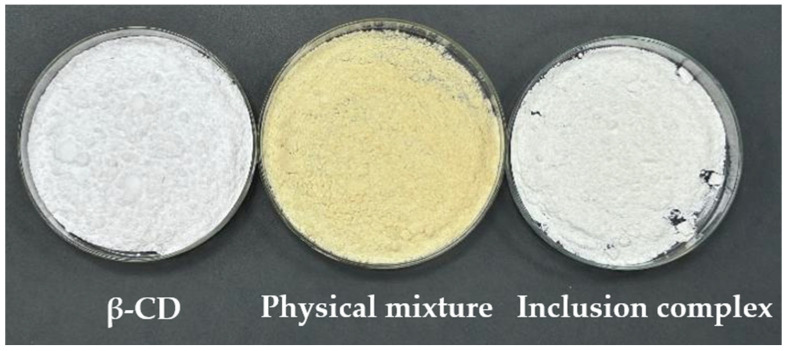
Apparent condition.

Figure 5 showed that the β-cyclodextrin powder was fine in texture, while the physical mixture and inclusion complexes were relatively hard. Moreover, the physical mixture was yellow in color, while both β-cyclodextrin and the inclusion complexes were white.

#### 3.4.2. Microscopic Analysis

**Figure 6:**
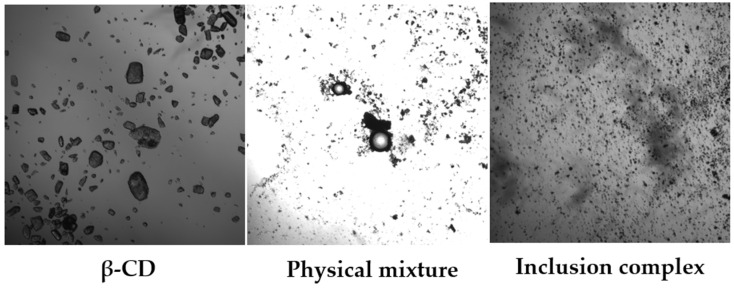
Microscopic images (50×).

Figure 6 shows that β-cyclodextrin presents regular transparent plate-like crystals. In the physical mixture of volatile oils, there are transparent plate-like crystals and oil droplets. In contrast, after the volatile oils are encapsulated, both of them present irregular opaque crystals. These results indicate that, compared with physical mixing, the volatile oils may have undergone changes after the encapsulation process, resulting in a change in the state of the substances.

#### 3.4.3. Fourier-Transform Infrared Spectroscopy (FT-IR) Analysis

**Figure 7:**
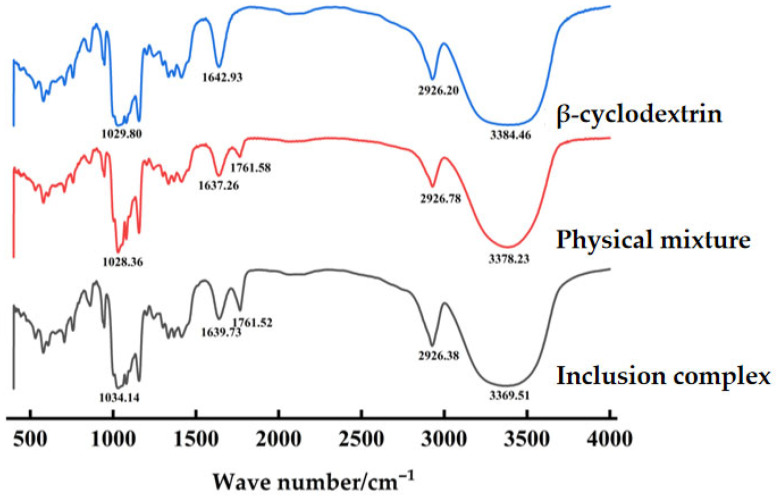
FT-IR scanning spectra.

Figure 7 illustrates that the infrared spectrum of β-cyclodextrin exhibits five characteristic peaks: the -OH stretching vibration peak at approximately 3384 cm^−1^, the -CH_2_ and -CH_3_ stretching vibration peaks at around 2926 cm^−1^, the OH bending vibration peak at about 1642 cm^−1^ (which may originate from adsorbed water or the hydroxyl groups of cyclodextrin), and the C–O–C stretching vibration peak at approximately 1029 cm^−1^. The physical mixture retains the characteristic peaks of the guest’s carbonyl group (1761 cm^−1^) and an independent O–H peak (3378 cm^−1^). In contrast, these features are further intensified in the inclusion complex, such as the enhancement of the guest’s carbonyl peak (1761 cm^−1^). Additionally, from β-cyclodextrin to the inclusion complex, the -OH stretching vibration peak undergoes a red shift (from 3384 cm^−1^ to 3369 cm^−1^), indicating that the hydrogen bonding of the hydroxyl groups is strengthened after encapsulation. The guest molecules enter the cavity of cyclodextrin and form new, stronger hydrogen bonds with the hydroxyl groups. This causes a decrease in the force constant of the O–H bond, lowering its vibrational frequency, and thus the peak position shifts to a lower wavenumber. This suggests that the guest molecules have entered the cyclodextrin cavity and interacted with the hydroxyl groups, leading to a rearrangement of the hydrogen bonding network.

#### 3.4.4. X-Ray Diffraction (XRD) Analysis

**Figure 8:**
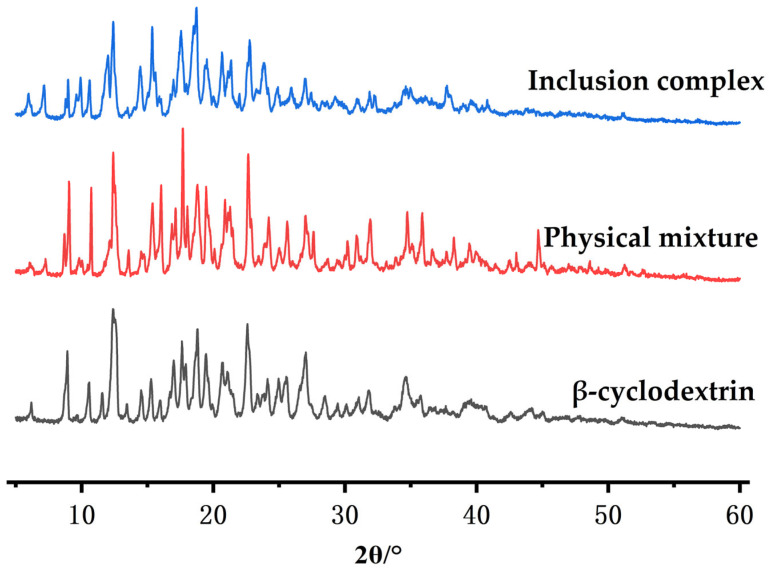
XRD pattern.

Figure 8 shows that β-cyclodextrin exhibits multiple distinct and strong diffraction peaks, mainly distributed within the 10° to 30° range of the 2θ spectrum. The typical peak positions are approximately 12.5°, 15.5°, 18.5°, 22.5°, 27.0°, etc. This is largely consistent with the diffraction pattern of the β-cyclodextrin crystal state, indicating that it has a regular cage-like or channel-like crystal structure. The diffraction peaks of the physical mixture basically retain all the characteristic peaks of β-cyclodextrin (positions are almost the same), but new small peaks appear in certain areas (such as around 20.0°), and the relative intensity of the peaks slightly changes. This may be attributed to the formation of the physical mixture—both components (β-cyclodextrin + volatile oil molecules) maintain their crystal structures, and the diffraction pattern is the simple superposition of the two crystal peaks. In the spectrum of the inclusion complex, the original sharp peaks of β-cyclodextrin mostly disappear or significantly weaken, and new broader diffraction peaks appear, with the original peak shape broadening and intensity decreasing. The overall pattern shows the characteristics of an amorphous state or a new crystal phase. This provides certain evidence for the formation of the complex.

#### 3.4.5. Differential Scanning Calorimetry (DSC)

**Figure 9:**
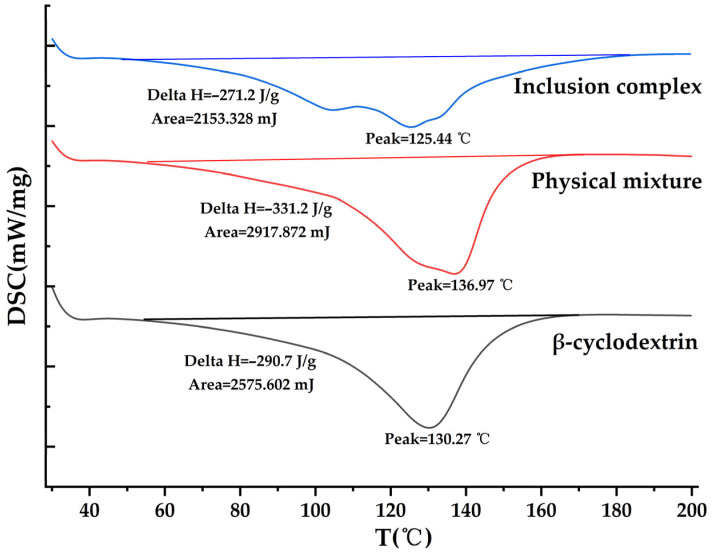
DSC.

As shown in Figure 9, the blank β-cyclodextrin shows an endothermic peak at 130.27 °C; the peak shape of the physical mixture is basically similar to that of the blank β-cyclodextrin; the spectrum of the inclusion compound has changed, with the endothermic peak shifting to 125.44 °C, the peak shape becoming wider, the heat enthalpy decreasing, and new shoulder peaks appearing. This indicates that some changes have occurred in the structure of the substance during the encapsulation process. As for the formation of the shoulder peaks, this might be due to the multi-step dissociation of the inclusion compound sample, and the inclusion compound may not completely dissociate in one step when heated. At the same time, the physical mixture is a sharp single peak without shoulder peaks, which also suggests that it might be due to the inhomogeneity of the encapsulation state.

#### 3.4.6. Phase-Solubility Studies

**Figure 10:**
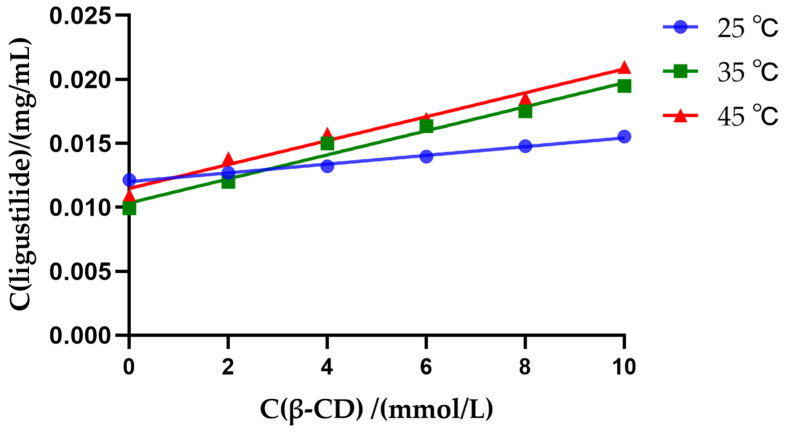
The dissolution curves of ligustilide at different temperatures.

The results of phase-solubility studies are shown in Figure 10 and Table 12. From the dissolution curves, it was calculated that the inclusion constants at 25, 35, and 45 °C were 0.0475, 0.1661, and 0.1488 L/mmol respectively. The Kc value was between 0.0475 and 0.1661 L/mmol, indicating that the ligustilide formed a medium-to-weakly strong inclusion complex with the host. The inclusion constant increased with the increase in temperature, which might be due to the decrease in intermolecular forces (van der Waals forces between the guest and the cavity, “hydrophobic bond forces” between the hydrophobic guest and the cavity, etc.). ΔH was all positive, indicating that the inclusion reaction was an endothermic process. At the three experimental temperatures, ΔG was all positive (4.59–7.54 kJ/mol), indicating that the inclusion process was non-spontaneous under standard conditions (1 mol/L). ΔG decreased first and then slightly increased with the increase in temperature, reaching the minimum value at 35 °C. This is because ΔG = ΔH − TΔS. When ΔH and ΔS are both positive, the increase in temperature makes the term (−TΔS) more negative, which is conducive to the reduction in ΔG (making the process easier to proceed); but when the temperature is further increased, ΔH and ΔS themselves may change (considered as constants in this analysis), or the inherent solubility of the guest may change, resulting in a change in Kc value (the Kc value slightly decreased at 45 °C), causing ΔG to rise again. The inclusion process is jointly determined by the positive ΔS (entropy-driven) and the positive ΔH (enthalpy unfavorable). Within the experimental temperature range, the term TΔS (entropy contribution) is greater than the term ΔH (enthalpy contribution), which is the main reason for ΔG to be negative (in non-standard conditions) or a small positive value. Therefore, hydrophobic interaction is probably the main driving force for the formation of the inclusion complex of ligustilide. Therefore, the inclusion of ligustilide is an endothermic process driven by entropy, with hydrophobic forces being dominant. A temperature of 35 °C is a more suitable inclusion temperature, at which the inclusion constant is the largest and the Gibbs free energy change is the smallest. At the same time, this also confirms our optimal process temperature condition, which is 30 °C.

#### 3.4.7. Molecular Docking

**Figure 11:**
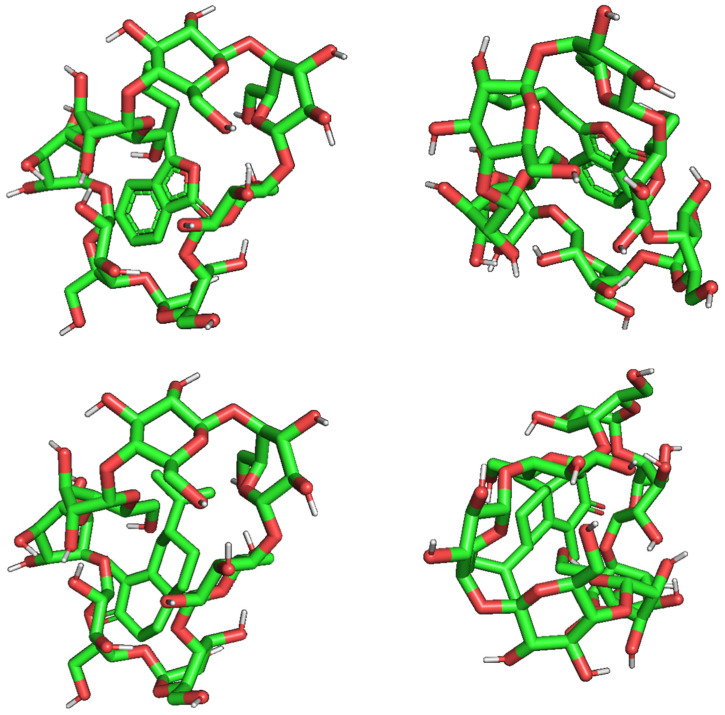
DSC: Top view and side view of the molecular docking of ligustilide with β-cyclodextrin molecule and α-cyperone with β-cyclodextrin molecule. Among them, the red frame represents “O”, the red frame represents “C”, and the white part represents “H”.

The results of molecular docking are shown in Figure 11 and Table 13. The results indicate that the lactone rings of ligustilide and α-cyperone deeply penetrate the cavity of β-cyclodextrin to form inclusion complexes. Moreover, the energy of the docking conformation is relatively low, suggesting that the inclusion complexes are relatively stable. Additionally, these two components form hydrogen bonds with some β-cyclodextrin molecules. Furthermore, the results also show that the binding of the compound to the β-cyclodextrin molecule is also affected by other intermolecular forces, such as van der Waals forces.

## 4. Discussion

This study conducted an HPLC-MS analysis on the volatile oil components of Ligusticum Chuanxiong–Vinegar cyperus rotundus. Two components, ligustilide and α-cyperone, are detected in the analysis. In the 2025 edition of the Chinese Pharmacopeia, ligustilide and α-cyperone are respectively selected as the detection components for the identification of Ligusticum Chuanxiong and Vinegar cyperus rotundus medicinal materials [36]. Furthermore, the study has found that ligustilide is one of the active ingredients in TianShu Capsules and may be involved in the drug’s efficacy in treating migraines [37]. Additionally, when studying the therapeutic effects of Xiongfu Dropping Pills, ligustilide and α-cyperone were selected as quality control components, and it was suggested that these two substances might be related to their ability to treat migraines [38]. In conclusion, we believe that ligustilide and α-cyperone may be the active components of the volatile oils of Ligusticum Chuanxiong and Vinegar cyperus rotundus. Therefore, these two components are selected as the indicative compounds for evaluating the encapsulation rate of the inclusion complex. This provides a basis for the quality control goals in the subsequent encapsulation experiments.

The experimental results show that when the concentration of β-cyclodextrin is increased by a factor of two, the inclusion rate actually decreases. This phenomenon may be caused by some physical and chemical factors: 1. Competitive hydration and colloid formation: The β-cyclodextrin molecules themselves are hydrophilic, and their outer surface is rich in hydroxyl groups. When the concentration is too high, a large number of β-cyclodextrin molecules will strongly combine with water molecules (hydration), consuming a large amount of “free water” in the system. The inclusion process of volatile oil also requires a water environment as a medium, and the reduction in free water may indirectly affect the kinetics of the inclusion process. 2. Formation of colloid aggregates: Excessive β-cyclodextrin molecules may aggregate with each other, forming larger colloid aggregates. The internal cavities of these aggregates may deform or be blocked due to intermolecular interactions, and the effective inclusion sites do not increase proportionally with the dosage. Volatile oil molecules are more likely to combine with the free monomeric β-cyclodextrin in the solution, and these aggregates become “ineffective” components [39]. 3. Disruption of phase equilibrium and change in precipitation mechanism: The inclusion process is a dynamic equilibrium: volatile oil + β-cyclodextrin ⇌ inclusion complex. At an appropriate ratio, the generated inclusion complex dissolves in water, and the reaction proceeds to the right. When β-cyclodextrin is over-concentrated to an extreme extent, a chemical ratio different from the original and with a lower solubility may form an inclusion complex.

Common inclusion materials for preparing Chinese herbal volatile oil inclusion complexes are β-cyclodextrin and hydroxypropyl-β-cyclodextrin. Experimental studies indicate that hydroxypropyl-β-cyclodextrin facilitates the dissolution of Chuanxiong volatile oil [40], but the stability of Chuanxiong volatile oil components in the inclusion complex is lower than that in β-cyclodextrin [41]. This is likely attributed to the increased water solubility of hydroxypropyl-β-cyclodextrin, which readily binds with water molecules in the air, thereby reducing the stability of Chuanxiong volatile oil components within it. Considering the need to control hygroscopicity in subsequent granule formulations, β-cyclodextrin was selected as the inclusion material.

Exploration and optimization of the Chuanxiong–Vinegar cyperus volatile oil inclusion process based on QbD principles necessitated simultaneous investigation of inclusion speed, inclusion time, inclusion temperature, and feed ratio. Following CMA parameter analysis, a single-factor experiment combined with a factorial design was employed. Inclusion speed was first examined as a single factor, followed by a factorial design (2^3^) for inclusion time, inclusion temperature, and feed ratio. This approach significantly reduced experimental runs while rapidly identifying key factors significantly affecting inclusion rate, elucidating their interactions, and enabling targeted optimization. This approach significantly reduced the number of experiments while enabling rapid identification of key factors significantly affecting the encapsulation rate. It also revealed the interactions between these factors and their impact on the encapsulation rate, allowing targeted optimization adjustments to achieve optimal encapsulation results [17]. However, the encapsulation temperature considered in this experiment represents only the preset temperature during the encapsulation process. In practice, the entire solution system undergoes temperature changes during volatile oil droplet addition. The potential loss of volatile oil during this process was not accounted for, which may be one reason the experimental encapsulation rate remained between 50% and 60%. Therefore, in the validation experiment, increasing the amount of volatile oil added reduced the proportion of volatile oil lost during this step, leading to an improvement in the encapsulation rate.

In this study, DSC reflects the thermodynamic changes in the samples. However, the influence of moisture on the β-cyclodextrin itself cannot be completely excluded. Thermogravimetric analysis (TGA), as a technique capable of real-time monitoring of sample mass changes, can precisely and quantitatively determine the moisture content in the β-cyclodextrin system, thereby making the attribution of thermal events clearer. Therefore, in future research, by combining DSC and TGA technologies, it will be possible to more accurately reflect the thermodynamic events of the guest molecules encapsulated by the β-cyclodextrin system.

## 5. Conclusions

1. Ligustilide and α-cyperone may serve as the initial indicator components in the encapsulation process of the mixed volatile oil of Ligusticum Chuanxiong–Vinegar cyperus Rotundus, but their representativeness in terms of encapsulation quality still requires further pharmacological evaluation;
2. Due to its excellent solubility, stability, and the ability to control the hygroscopicity in subsequent formulations, β-cyclodextrin was selected as the preferred encapsulation material;
3. The combination of single-factor and factorial design with the QbD principle can effectively optimize the encapsulation process, determine the key factors, and reduce the experimental workload. It also demonstrates the feasibility and practicality of this concept in process optimization;
4. The formation of inclusion complexes generally leads to a series of changes in physical and chemical properties. These changes can be demonstrated by methods such as Fourier-transform infrared spectroscopy (FT-IR), X-ray diffraction (XRD), and differential scanning calorimetry (DSC), and thus can be used as evaluation indicators for the inclusion process.

## Figures and Tables

**Table 1:** The risk assessment results of PCMPs in the inclusion experiment.

Category	Method Parameters	S	O	D	RPN	RPN Level	Explanation
Inclusion material	β-cyclodextrin	5	8	7	280	High	The risk of affecting the inclusion rate is relatively high.
	Hydroxypropyl-β-cyclodextrin	5	8	7	280	High	
Environment	Temperature	6	8	3	144	High	The inclusion temperature has a significant impact on the volatile components.
	Environmental humidity	3	1	4	12	Low	
Equipment	Stirring speed	5	6	3	90	Middle	The method of stirring may affect the degree of uniform mixing.
	Stirring method	5	2	3	30	Low	
Process	Inclusion temperature	9	9	3	243	High	It directly affects the encapsulation effect and poses a high risk.
	The ratio of volatile oil to inclusion materials	9	9	5	405	High	
Other factors	Operator	2	2	6	24	Low	The experimental equipment is of high precision, and all the reagents are of qualified quality, with relatively low risks.
	Batch of materials	4	2	3	24	Low	
	Precision of analytical instruments	7	1	2	14	Low	

**Table 2:** Combined rotational speed investigation experiment combination (n = 3).

Group	Doubled Concentration of β-Cyclodextrin Solution	Inclusion Time/h	Temperature/°C	Stirring Speed/rpm
1	6	1	Room Temperature	300
2	6	1	Room Temperature	800

**Table 3:** Factorial design parameter levels for volatile oil inclusion test.

Factor	Level	
	−1	+1
A: β-Cyclodextrin solution ratio	6	10
B: Inclusion time/h	1	3
C: Temperature/°C	30	50

**Table 4:** Factorial design experiment combination.

Std ^1^	Block	Run	β-Cyclodextrin Solution Ratio	Inclusion Time/h	Temperature/°C
1	Block1	4	6	1	30
2	Block1	5	10	1	30
3	Block1	6	6	3	30
4	Block1	1	10	3	30
5	Block1	8	6	1	50
6	Block1	2	10	1	50
7	Block1	3	6	3	50
8	Block1	7	10	3	50
9	Block2	10	6	1	30
10	Block2	12	10	1	30
11	Block2	14	6	3	30
12	Block2	15	10	3	30
13	Block2	16	6	1	50
14	Block2	9	10	1	50
15	Block2	3	6	3	50
16	Block2	11	10	3	50

^1^ Std.—the order in which the experimental design is labeled according to standard rules.

**Table 5:** Identification results of major chemical constituents in Chuanxiong–Vinegar cyperus essential oil.

Number	Observed RT (min)	Component Name	Parent Ion	Mass Error (ppm)
1	9.31	(1R,5R)-2-methyl-5-prop-1-en-2-ylcyclohex-2-en-1-ol	[M+H] + 153.1261	−8.4
2	21.43	(-)-Cyperenoic acid	[M+H] + 249.1839	−4
3	16.11	1-(2,4-dimethylphenyl)propan-1-one	[M+H] + 163.1107	−6.3
4	7.3	2-(butyn-2-ylidene)-delta3-dihydrofuran [5-spiro-2′]tetrahydrofuran	[M+H] + 179.1057	−5.3
5	12.58	5,7-diethyldeca-5,6-dien-3-yne	[M+H] + 191.1796	1.2
6	7.5	prop-2-en-1-yl 2-phenoxyacetate	[M+H] + 193.085	−4.6
7	19.16	(2′Z,3S,8′R)-2′-butylidene-8′-propylspiro [2-benzofuran-3,9′-3-oxatricyclo [5.2.2.01,5]undec-5-ene]-1,4′-dione	[M+H] + 379.1903	−0.2
8	13.07	(4aR,6R,8aS)-4a-hydroxy-4,8a-dimethyl-6-prop-1-en-2-yl-5,6,7,8-tetrahydro-1H-naphthalen-2-one	[M+H] + 235.1682	−4.4
9	15.36	2,4-Di-tert-butylphenol	[M+H] + 207.1732	−5.4
10	11.49	1,1-diphenylpentan-1-ol	[M+H] + 165.1262	−7
11	14.43	Isocurcumenol	[M+H] + 235.1684	−3.8
12	16.55	Isokobusone	[M+H] + 223.1682	−4.6
13	5.65	(3E)-3-butylidene-6,7-dihydroxy-4,5,6,7-tetrahydro-2-benzofuran-1-one	[M+H] + 225.111	−5
14	17.82	(16Z)-6-butyl-16-butylidene-5,15-dioxapentacyclo [9.5.2.01,13.02,10.03,7]octadeca-3(7),12-diene-4,14-dione	[M+H] + 383.221	−1.8
15	16.87	(Z)-6,8,7,3-Diligustilide	[M+H] + 353.1753	1.5
16	15.35	(S)-4-isopropenylcyclohex-1-enecarbaldehyde	[M+H] + 151.1103	−9.2
17	9.18	3-butylidene-4,5-dihydro-2-benzofuran-1-one	[M+H] + 207.1008	−3.6
18	7.69	Senkyunolide K	[M+H] + 209.1165	−3.6
19	23.43	Senkyunolide P	[M+H] + 383.2212	−1.3
20	27.69	Senkyunone	[M+H] + 327.2311	−2.2
21	14.17	(4aS,7R)-7-isopropenyl-1,4a-dimethyl-4,4a,5,6,7,8-hexahydronaphthalen-2(3H)-one	[M+H] + 219.1733	−4.6
22	11.59	[(1R,2S,4R)-1,7,7-trimethyl-2-bicyclo [2.2.1]heptanyl] acetate	[M-H]-195.138	−5.4
23	5.63	(3E,6R,7R)-3-butylidene-6,7-dihydroxy-4,5,6,7-tetrahydro-2-benzofuran-1-one	[M-H]-223.0965	−4.8
24	9.9	(Z)-3-butylidene-5-hydroxy-2-benzofuran-1-one	[M-H]-203.0702	−6
25	16.87	(Z)-6,8′7,3′-Diligustilide	[M-H]-379.1912	−0.7
26	19.29	(Z,Z′)-Diligustilide	[M-H]-379.1918	0.7
27	27.06	13-methylpentadecanoic acid	[M-H]-255.232	−3.6
28	29.22	14-methylhexadecanoic acid	[M-H]-269.2479	−2.6
29	12.52	3-butyl-4,7-dihydroxy-2-benzofuran-1(3H)-one	[M-H]-221.0812	−3.2
30	6.11	3-n-Butyl-3-hydroxy-4,5,6,7-tetrahydro-6,7-dihydroxy phthalide	[M-H]-241.1068	−5.4
31	9.05	3-butyl-4-hydroxy-2-benzofuran-1(3H)-one	[M-H]-205.0863	−3.7
32	26.52	methyl (9Z,12Z)-octadeca-9,12-dienoate	[M-H]-293.2477	−2.9
33	12.77	(4aR,7R)-7-isopropenyl-1,4a-dimethyl-4,4a,5,6,7,8-hexahydronaphthalen-2(3H)-one	[M-H]-217.1596	−1
34	9.28	4-ethyl-2-methoxyphenol	[M-H]-165.0911	−5.8
35	16.53	Cyperolone	[M-H]-235.1693	−4.3
36	13.48	Epoxydihydrolinalool	[M-H]-171.1379	−7
37	34.83	Ethylisooctadecanoate	[M-H]-311.2948	−2.3
38	12.51	1,2-dimethoxy-4-prop-2-enylbenzene	[M-H]-177.0911	−5.8
39	6.99	2-methyl-1-phenylpropan-1-one	[M-H]-147.0801	−9.6
40	16.7	(Z)-3-butylidene-4,5-dihydro-2-benzofuran-1-one	[M-H]-189.0911	−5.5
41	16.68	Ligustilide dimer, E-232	[M-H]-379.1912	−0.8
42	25.06	(9Z,12Z)-octadeca-9,12-dienoic acid	[M-H]-279.232	−3.4
43	11.06	methyl 2-pentanoylbenzoate	[M-H]-219.1014	−5.9
44	27.02	Myricanone	[M-H]-355.1578	7.5
45	11.48	nonanoic acid	[M-H]-157.1223	−7.1
46	27.99	(9Z)-octadec-9-enoic acid	[M-H]-281.2479	−2.7
47	7.24	4-isopropenylcyclohex-1-enecarbaldehyde	[M-H]-149.096	−7.9
48	12.57	Senkyunolide G	[M-H]-207.1008	−8.8
49	5.33	Senkyunolide J	[M-H]-225.1122	−4.5
50	5.33	Senkyunolide R	[M-H]-239.0916	−3.8
51	31.24	octadecanoic acid	[M-H]-283.2634	−2.9
52	16.08	Sugebiol	[M-H]-235.1691	−5.2
53	10.94	Sugetriol	[M-H]-251.1639	−5.4
54	15.62	undecanoic acid	[M-H]-185.1533	−7.8
55	3.93	4-hydroxy-3-methoxybenzoic acid	[M-H]-167.0339	−6.4

**Table 6:** Experimental results of combined rotational speed.

Group	Combined Rotational Speed/rpm	Sample Weight/g	Ligustilide Content/mg	α-Cyperone Content/mg
1	300	3.00012	11.53124	0.04369
2	300	3.00012	11.52805	0.04370
3	300	3.00023	11.57773	0.04397
4	800	3.00023	11.59214	0.04441
5	800	3.00015	11.50806	0.04391
6	800	3.00015	11.48639	0.04395

**Table 7:** Factorial experiment design and results.

Std	Run	A: β-Cyclodextrin Solution Ratio	B: Inclusion Time/h	C: Temperature/°C	Ligustilide Inclusion Rate/%	α-Cyperone Inclusion Rate/%
1	4	6	1	30	56.68	56.44
2	5	10	1	30	42.29	38.12
3	6	6	3	30	68.99	74.28
4	1	10	3	30	55.93	43.92
5	8	6	1	50	63.61	68.47
6	2	10	1	50	49.13	44.59
7	3	6	3	50	44.69	68.43
8	7	10	3	50	44.36	41.86
9	10	6	1	30	58.19	61.83
10	12	10	1	30	37.19	39.66
11	14	6	3	30	59.1	82.87
12	15	10	3	30	58.66	45.49
13	16	6	1	50	59.13	66.81
14	9	10	1	50	43.3	54.13
15	3	6	3	50	41.99	74.36
16	11	10	3	50	38.75	44.09

**Table 8:** Analysis of variance results for ligustilide inclusion rate.

Source of Variation	Sum of Squares	Degrees of Freedom	Mean Square	F-Value	*p*-Value
Block	53.91	1	53.91		
Model	1147.03	4	286.76	12.88	0.0006
A: β-Cyclodextrin solution ratio	428.18	1	428.18	19.23	0.0014
B: Inclusiontime/h	0.5439	1	0.5439	0.0244	0.8789
C: Inclusiontemperature/°C	169.46	1	169.46	7.61	0.0202
BC	548.85	1	548.85	24.65	0.0006
Residual	222.63	10	22.26		
Cor total	1423.57	15			

**Table 9:** Analysis of variance results for α-cyperidone inclusion rate.

Source of Variance	Sum of Squares	Degrees of Freedom	Mean Square	F-Value	*p*-Value
Block	68.6	1	68.6		
Model	2888.61	4	722.15	34.36	<0.0001
A: β-Cyclodextrin solution ratio	2540.92	1	2540.92	120.9	<0.0001
B: Inclusiontime/h	127.97	1	127.97	6.09	0.0332
C: Inclusiontemperature/°C	25.33	1	25.33	1.21	0.298
BC	194.39	1	194.39	9.25	0.0124
Residual	210.17	10	21.02		
Cor total	3167.37	15			

**Table 10:** Shapiro–Wilk test for normal distribution.

Factor	W	*p*-Value	Passed Normality Test (alpha = 0.05)
Ligustilide inclusion rate	0.9256	0.2076	Yes
α-Cyperone inclusion rate	0.9120	0.1225	Yes

**Table 11:** Design space verification experiments and results (n = 2).

Group	β-Cyclodextrin Multiplication	Inclusion Time/h	Inclusion Temperature/°C	Ligustilide Inclusion Rate/%	95% Confidence Interval for Ligustilide	α-Cyperone Inclusion Rate/%	95% Confidence Interval for α-Cyperone
1	6	3	30	64.74	57.39~74.29	75.18	66.03~82.45
2	6	2	35	63.18	49.76~66.59	73.32	60.38~76.73
3	6	2.6	33	63.15	52.67~70.12	72.97	62.99~79.95
4	6	2.5	30	64.24	53.91~71.74	71.33	62.43~79.74
5	6	2.75	32	63.49	53.87~71.74	76.89	63.71~81.08

**Table 12:** Thermodynamic analysis.

Temperature/°C	Regression Equation	Kc/L·mmol^−1^	ΔH/KJ·mol^−1^	ΔG/KJ·mol^−1^	ΔS/J·mol^−1^·K^−1^
25	y = 0.0003x + 0.012 R^2^ = 0.9920	0.0475	39.18	7.54	106.00
35	y = 0.0009x + 0.0103 R^2^ = 0.9782	0.1661		4.59	
45	y = 0.0009x + 0.0115 R^2^ = 0.9827	0.1488		5.04	

**Table 13:** The molecular docking results of β-cyclodextrin with ligustilide and α-cyperone.

Component	Binding Energy
Ligustilide	−4.09
α-Cyperone	−4.18

## Data Availability

The original contributions presented in this study are included in the article. Further inquiries can be directed to the corresponding author.

## Abbreviations

The following abbreviations are used in this manuscript:

QbD	Quality by Design
HPLC-MS	High-performance liquid chromatography–tandem mass spectrometry
FT-IR	Fourier-transform infrared spectroscopy
FDA	Food and Drug Administration
UPLC-Q-TOF/MS	Ultra-performance liquid chromatography–quadrupole time-of-flight mass spectrometry
CQAs	Critical quality attributes
CMPs	Critical monitoring parameters
SMPs	Secondary monitoring parameters
MODR	Operable design region
DSC	Differential scanning calorimetry
TGA	Thermogravimetric analysis
XRD	X-ray diffraction
BPI	Base peak ionization spectra
